# The Asia Pacific Cardiovascular Research (ASPIRE) Network: Facilitating and Advancing Regional Cardiovascular Clinical Trials

**DOI:** 10.1016/j.jacasi.2025.05.019

**Published:** 2025-08-29

**Authors:** Clement Lau, Philip D. Adamson, Nicholas Chew, Gemma Figtree, Alan Fong, Diana HP Foo, Gary C.H. Gan, Wael Al Mahmeed, Chien-Yi Hsu, Chun-Yao Huang, Young-Hoon Jeong, Takashi Kajiya, Dharmaraj Karthikesan, Yongcheol Kim, Rungroj Krittayaphong, Chi-Hang Lee, Jiunn-Lee Lin, Siti Elkana Nauli, Seitaro Nomura, Duk-Woo Park, Leonardo Paskah Suciadi, Arintaya Phrommintikul, A Mark Richards, Anwar Irawan bin Ruhani, Chun-Che Shih, Supanee Sinphurmsukskul, Bambang Budi Siswanto, Indah Sukmawati, Sock Hwee Tan, Asha Thanabalan, Richard Troughton, Kenichi Tsujita, Wei Ting Wang, Bambang Widyantoro, Creany Wong, Satoshi Yasuda, Henry Yau, Kai-Hang Yiu, Akhmal Yusof, Mark Y. Chan, Derek J. Hausenloy

**Affiliations:** a Duke-National University of Singapore Medical School, Singapore; b National Heart Research Institute Singapore, National Heart Centre, Singapore; c Christchurch Heart Institute, University of Otago Christchurch, New Zealand; d Department of Medicine, University of Otago, Christchurch, New Zealand; e Department of Cardiology, National University Heart Centre, Singapore; f Department of Medicine, Yong Loo Lin School of Medicine, National University of Singapore, Singapore; g Faculty of Medicine and Health, University of Sydney, Sydney, NSW, Australia; h Sarawak Heart Centre, Kota Samarahan, Malaysia; i Clinical Research Centre, Sarawak General Hospital, Kuching, Malaysia; j Department of Cardiology, Westmead Hospital, Sydney, New South Wales; k Department of Cardiology, Blacktown Hospital, Sydney, New South Wales; l Heart, Vascular and Thoracic Institute, Cleveland Clinic Abu Dhabi, Abu Dhabi, United Arab Emirates; m Division of Cardiology and Cardiovascular Research Center, Department of Internal Medicine, Taipei Medical University Hospital, Taipei, Taiwan; n Division of Cardiology, Department of Internal Medicine, School of Medicine, College of Medicine, Taipei Medical University Taipei, Taiwan; o Taipei Heart Institute, Taipei, Taiwan; p CAU Thrombosis and Biomarker Center, Chung-Ang University Gwangmyeong Hospital, Gwangmyeong, South Korea; q Department of Internal Medicine, Chung-Ang University College of Medicine, Seoul, South Korea; r Department of Cardiology, Tenyoukai Central Hospital, Kagoshima, Japan; s Department of Cardiology, Sultanah Bahiyah Hospital, Malaysia; t Yonsei University College of Medicine and Cardiovascular Center, Yongin Severance Hospital, Yongin, South Korea; u Division of Cardiology, Department of Medicine, Siriraj Hospital, Mahidol University in Bangkok, Thailand; v Shuang-Ho Hospital Cardiovascular Center, Taipei Medical University, Taipei, Taiwan; w Cardiology and Vascular Department, Tangerang District Hospital, Indonesia; x Department of Cardiovascular Medicine, The University of Tokyo, Tokyo, Japan; y Department of Frontier Cardiovascular Science, The University of Tokyo, Tokyo, Japan; z Division of Cardiovascular Medicine, Asan Medical Center, Seoul, Korea; aa Siloam Heart Institute, Siloam Hospitals Kebon Jeruk, Jakarta, Indonesia; bb Clinical Research Department of Siloam Group, Banten, Indonesia; cc Medical School of Pelita Harapan University, Banten, Indonesia; dd Division of Cardiology, Department of Internal Medicine, Faculty of Medicine, Chiang Mai University, Chiang Mai, Thailand; ee Department of Cardiology, Tengku Ampuan Afzan Hospital, Kuantan, Pahang, Malaysia; ff Heart Failure and Transplant Cardiology, King Chulalongkorn Memorial Hospital, Thai Red Cross Society, Bangkok, Thailand; gg Indonesia Medical Education and Research Institute, Cardiovascular Research Center National Cardiovascular University Indonesia; hh Siloam Hospitals Lippo Village, Tangerang, Indonesia; ii Universitas Pelita Harapan, Tangerang, Indonesia; jj Cardiovascular DiseasE National Collaborative Enterprise (CADENCE), Singapore; kk Clinical Research Malaysia (CRM), Kuala Lumpur, Malaysia; ll Department of Cardiovascular Medicine, Kumamoto University, Kumamoto, Japan; mm Division of Cardiology, Department of Internal Medicine, Taipei Veterans General Hospital, Taipei, Taiwan, R.O.C; nn School of Medicine, National Yang-Ming Chiao-Tung University, Taipei, Taiwan, R.O.C; oo Institute of Clinical Medicine, National Yang-Ming Chiao-Tung University, Taipei, Taiwan, R.O.C.; pp Department of Cardiology and Vascular Medicine, Faculty of Medicine Universitas Indonesia – National Cardiovascular Center Harapan Kita, Jakarta, Indonesia; qq Clinical Trials Centre, The University of Hong Kong, Hong Kong; rr Department of Cardiovascular Medicine, Tohoku University, Sendai, Japan; ss Division of Cardiology, Department of Medicine, The University of Hong Kong, Hong Kong; tt Cardiovascular & Metabolic Disorders Program, Duke-National University of Singapore Medical School, Singapore; uu The Hatter Cardiovascular Institute, University College London, London, United Kingdom

**Keywords:** APAC trials, ASPIRE, cardiovascular trials, clinical trials, clinical trials network

**Figure:**
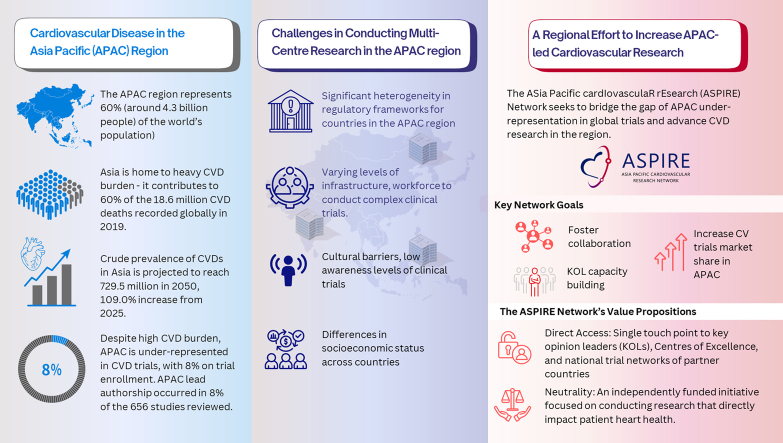

The Asia-Pacific (APAC) region comprises a diverse array of countries and is home to approximately 4.6 billion people—accounting for nearly 60% of the global population. Cardiovascular diseases (CVDs) are the leading cause of death and disability in the APAC region, with a rising burden particularly in the low- to middle-income countries. In 2021, it is estimated that CVDs contributed to nearly 44% of deaths in the region.^1^

Despite this significant health burden, there is a marked under-representation of patients from the APAC region in international multicenter cardiovascular clinical trials, most of which are conducted in Western countries. Given that randomized controlled trials form the cornerstone of evidence-based practice and clinical guidelines, this lack of representation has important implications for the applicability and translation of effective diagnostic, preventive, and therapeutic measures into clinical practice across the region. To address this gap, we have established the Asia Pacific Cardiovascular Research (ASPIRE) Network, a collaborative effort among countries in the APAC region to facilitate and advance regional cardiovascular clinical trials through a collaborative approach.

The under-representation of APAC populations in cardiometabolic disease trials is particularly noteworthy.^2^ Between 2000 and 2020, only 8% of participants in global heart failure trials were recruited from Asia and Oceania, despite over 50% of the world’s patients with heart failure residing in these regions.^3^ In a systematic review by Azzopardi et al,^4^ examining 656 cardiovascular randomized controlled clinical trials in diabetes and obesity from 3 major leading medical journals between 2011 and 2020, only 8.3% of participants were identified as Asian, with just 7.7% of enrolment occurring in APAC. Although there has been a significant increase in the proportion of patients of Asian ethnicity enrolled during this period, the increase was modest (1%-2%) and trial participant-to-prevalence ratio, a measure of the proportion of APAC participants in the clinical trials comparable to the global prevalence, remained disappointingly low (participant-to-prevalence ratio <0.30) across all cardiometabolic diseases. Further, APAC-led authorship occurred in only 8% of included trials, correlating with regional participant enrolment. Whereas the study focused on high-impact journals and may not capture the full spectrum of trials globally, the findings underscore the need to address ethical, regulatory, and racial diversity challenges that hinder the conduct of clinical trials and patient recruitment in the region.

Even with robust clinical trial evidence supporting the use of drugs or medical devices in phase 3 studies and their endorsement in international clinical guidelines, translation into clinical practice can be challenging in the APAC region. A major obstacle lies in the heterogeneity of regulatory frameworks across the region, although steps toward harmonization are emerging. A lack of representative data on the local population often delays the approval of therapeutics by regulatory authorities and diminishes the external validity of seminal findings from randomized controlled trials, contributing to clinical decision inertia, which in turn hinders optimal therapeutic delivery. Regional differences in CVD prevalence and associated cardiovascular risk factors further complicate implementation of therapeutics. Compared to the rest of the region, stroke, hypertension, and lean diabetes are much more common in Eastern and Southeastern Asia.^5^ Additionally, body mass index thresholds for overweight and obesity differ in Asian populations with a body mass index of ≥23 kg/m^2^ defined as overweight and ≥25 kg/m^2^ defining obesity. Further, the varying levels of health literacy across APAC also affect trial recruitment, with patient willingness to participate closely linked to their understanding of research.

Pharmacogenomic and physiological differences also influence drug efficacy and safety. Compared to Western patients, Asians have heightened responses to some therapeutic drugs. For instance, Asian patients typically achieve lipid targets with lower doses of statins^6^ with corresponding black box warnings cautioning higher risk of off-target effects with higher statin doses. Asians also have an increased risk of bleeding with aspirin for primary prevention of CVD and P2Y_12_ antagonists in secondary prevention.^7^ Consequently, anticoagulation protocols in the region often specify a lower international normalized ratio therapeutic range due to higher perceived risk in intracranial bleeding rates in East Asia.^7^ Variations in Asians also exist in defining normality for cardiac structure and function.^8^ Furthermore, there are discernible differences in the presentation of cardiovascular diseases: for example, heart failure in Asians is diagnosed at younger age, with higher prevalence of diabetes, and lower ischemic etiology.^9^

Age-standardized cardiovascular mortality varies significantly across Asia. This is likely due to complex multiple interactions between individual (genetic, metabolic, behavioral, and lifestyle) and population (socioeconomic, cultural, environmental, and health care) factors. These disparities affect not only clinical event risk and access to health care, but also the real-world effectiveness of interventions. In addition, trials conducted in Western countries often reflect higher standards of care and resource availability, which may not be generalizable to the APAC region with highly heterogenous background treatment and standards of clinical care.^10^

Conducting large-scale randomized controlled trials effectively in the APAC region can be challenging due to unique logistical and infrastructural barriers. These include linguistic diversity, a wide range of nonurban settings across a large geographical area, underdeveloped transportation infrastructure, limited access to medical devices and technologies, and variability in clinical research expertise and biobanking capabilities.

Nonetheless, the APAC region has much to learn from successful models established elsewhere: for example, the European Clinical Research Infrastructure Network, which was established to provide a unified framework to support clinical trials across Europe. Similarly, a region-wide effort to unite clinical research and infrastructure in APAC could provide the foundation for meaningful change. Increasing Asian patient participation in clinical trials begins with bringing together clinicians and clinical scientists from across this diverse region. The incentive is clear; greater inclusion in APAC-led and authored clinical studies that reflect local population and clinical contexts. This requires an innovative and visionary effort to transform how trials are conducted, spearheaded by leading academics in the region.

This vision has culminated in the formation of the ASPIRE network, an independently funded initiative aimed at fostering cardiovascular clinical trials across the APAC region. The ASPIRE network was officially launched at the Asia Pacific Society of Cardiology Congress in April 2025. The ASPIRE network brings together leading experts from countries/areas across the APAC region including Singapore, Malaysia, Hong Kong, Taiwan, Japan, South Korea, Australia, New Zealand, Indonesia, United Arab Emirates, and Thailand and is open to new members as it expands. ASPIRE network members will be invited to form a steering committee to oversee activities of the ASPIRE network, and regular network meetings will enable members to discuss projects and initiatives for the collaboration. The ASPIRE network will leverage existing national and regional trial infrastructures. Notable examples include the national cardiovascular clinical trials network established in Singapore by the Cardiovascular Disease National Collaborative Enterprise (CADENCE) and the national clinical trials network established in Malaysia by Clinical Research Malaysia, both of which provide a centralized platform for the design, implementation, and conduct of cardiovascular clinical trials and streamline the regulatory and administrative processes to facilitate academic and industry collaboration. By integrating such platforms, ASPIRE seeks to build a robust, interconnected ecosystem for conducting cardiovascular research in the region.

The primary mission of the ASPIRE network is to foster collaboration among APAC leaders in cardiovascular research and accelerate knowledge discovery and translation through increased numbers of coordinated multicenter clinical trials and research studies conducted in the APAC region (Figure 1). The region has already demonstrated the fastest growth in global clinical trial activity between 2010 and 2020. By harnessing this momentum, the ASPIRE network hopes to attract increased investment through industry-sponsored trials and to support multinational investigator-initiated trials, both academically funded and hybrid academic-industry collaborations across the APAC region. This collaborative approach will help to drive research, generating much-needed high-quality local and regional data that will help better inform local clinical practice.

**Figure 1:**
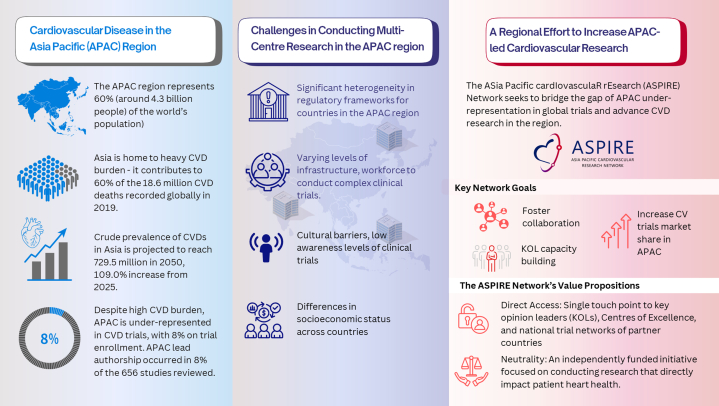
ASPIRE Network’s Mission to Drive Regional CV Research in the APAC Region The Asia Pacific Cardiovascular Research (ASPIRE) Network’s mission to drive regional cardiovascular research in the Asia-Pacific (APAC) region. CVD = cardiovascular disease; KOL = key opinion leader.

The ASPIRE network also seeks to address the disparity in clinical trials’ conduct by facilitating the sharing of expertise among APAC countries/areas. Connecting well-experienced trialists through this network will help develop and enhance early career or less experienced researchers and importantly it will provide a platform to mentor future clinical academics or scientists. Whereas the network is regionally focused, it will also aim to establish stronger ties with global partners and contribute to international trial consortia. Enhanced representation of APAC research at international conferences and high-impact journals will bolster the region’s standing and reputation for conducting high-quality clinical trials and advocate for greater inclusion of Asian participation in large-scale clinical trials.

Whereas the ASPIRE network is in its early stages, it marks a vital step in a broader, multifaceted approach to garner interest and foster investment in research from industry and government. Key strategies should include increasing diversity in patient recruitment, patient education about clinical trials, expanding access to research in less urban settings, and advocating for the inclusion of ethnicity data in trial reporting. Tailored regional recruitment strategies and quota systems set by regulatory bodies may further support equitable participation.

Moreover, the rapid evolution of artificial intelligence offers promising tools to transform the way that clinical research is conducted. Artificial intelligence can be used at various levels—from patient identification and enrolment to data acquisition analysis and interpretation—ultimately enabling more efficient and cost-effective research, even in resource-limited settings.

In summary, given the high cardiovascular burden, and under-representation in contemporary trials, the APAC region holds vast promise in serving as a platform for future clinical trials. With growing recognition within the cardiovascular academic community, there is an opportunity to overcome long-standing barriers that are limiting clinical trials and drive a new era of regional collaboration. By uniting leading researchers, health care professionals, research centers, and industry partners, the ASPIRE network is poised to lead cutting-edge research and generate meaningful insights to improve cardiovascular outcomes across the APAC region.

## Funding Support and Author Disclosures

Dr Richards has received speaker honoraria and/or advisory board fees and/or research grants and/or research support in-kind from Roche Diagnostics, Abbott Laboratories, Thermo Fisher, Novartis, AstraZeneca and Novo Nordisk; and holds the New Zealand Heart Foundation Chair in Cardiovascular Studies. Dr Tsujita has received research grants from PPD-Shin Nippon Biomedical Laboratories and Alexion Pharmaceuticals; scholarship funds from Abbott Medical, Bayer, Boehringer Ingelheim, Daiichi-Sankyo, ITI, Ono Pharmaceutical, Otsuka Pharmaceutical, and Takeda Pharmaceutical; has been affiliated with the endowed department from Abbott Medical, Boston Scientific, Cardinal Health, Fides-ONE, Fukuda Denshi, GM Medical, ITI, Japan Lifeline, Kaneka Medix, Medical Appliance, Medtronic, Nipro, and Terumo; and has received honoraria from Abbott Medical, Amgen, AstraZeneca, Bayer, Daiichi-Sankyo, Medtronic, Kowa, Novartis Pharma, Otsuka Pharmaceutical, Pfizer, and Janssen Pharmaceutical. Dr Jeong has received honoraria for lectures from Daiichi-Sankyo, Sanofi-Aventis, Han-mi Pharmaceuticals, and Daewoong Pharmaceuticals; and research grants or support from Samjin Pharmaceuticals, Hanmi Pharmaceuticals, Yuhan Pharmaceuticals, Biotronik Korea, and U and I Corporation. Dr Chan has received significant past research funding from AstraZeneca to conduct clinical trials in the APAC region; and salary support from a National Medical Research Council Clinician-Scientist Award-Senior Category (MOH-000280-00). Prof Hausenloy has received support from the Duke-NUS Signature Research Programme funded by the Ministry of Health, Singapore Ministry of Health’s National Medical Research Council under its Singapore Translational Research Investigator Award (MOH-STaR21jun-0003) and the Cardiovascular Disease National Collaborative Enterprise (CADENCE) National Clinical Translational Program (MOH-001277-01); consultant fees from Faraday Pharmaceuticals Inc and Boehringer Ingelheim International GmbH; honoraria from Servier; and research funding from AstraZeneca, Merck Sharp & Dohme, and Novo Nordisk. All other authors have reported that they have no relationships relevant to the contents of this paper to disclose.
